# Evaluation of the Outcomes and Complications of Uterine Artery Embolization Using the GELFOAM Technique in Patients With Ectopic Pregnancy Located in the Cervix and Ectopic Pregnancies Resulting From Previous Cesarean Section

**DOI:** 10.1002/hsr2.71677

**Published:** 2025-12-21

**Authors:** Hossein Hemmati, Roya Faraji Darkhaneh, Mohaddseh Khaligh Dargahi, Mohaya Farzin, Sara Sharifnia, Habib Eslami Kenarsari, Zoubin Souri

**Affiliations:** ^1^ Department of Vascular Surgery, Razi Clinical Development Unit Guilan University of Medical Sciences Rasht Iran; ^2^ Razi Clinical Research Development Unit, Razi Hospital Guilan University of Medical Sciences Rasht Iran; ^3^ Department of Gynecology, School of Medicine, Reproductive Health Research Center, Alzahra Hospital Guilan University of Medical Sciences Rasht Iran; ^4^ Department of Radiology, School of Medicine, Poursina Hospital Guilan University of Medical Sciences Rasht Iran

**Keywords:** ectopic pregnancy, gel foam, uterine artery embolization

## Abstract

**Background and Aims:**

To evaluate outcomes and adverse events of uterine artery embolization (UAE) using the GELFOAM technique for cervical and cesarean scar ectopic pregnancies at a single tertiary center.

**Methods:**

We conducted a descriptive cross‐sectional case series over 1 year. Maternal age, β‐hCG levels, mass size, obstetric history, complications, and treatment success were abstracted from records. Descriptive statistics are presented as mean ± SD for approximately normally distributed variables and median (IQR) for skewed variables.

**Results:**

Among 41 patients, uterine preservation was achieved in 40 (97.6%), with no procedure‐specific complications and one non‐specific postoperative hematoma (2.4%). The single failure occurred in a delayed presentation and required further surgical intervention. For uterine preservation (40/41), the 95% confidence interval for the success proportion is 87.4%–99.6% (Wilson).

**Conclusion:**

UAE with GELFOAM appears to be an effective, fertility‐preserving option for cervical and cesarean‐scar ectopic pregnancy in selected patients. Larger studies with longer follow‐up are warranted.

## 1 Introduction

Ectopic pregnancy (EP) refers to implantation of a fertilized ovum outside the uterine cavity and occurs in about 2% of all pregnancies [1, 2, 3]. Approximately 97% of ectopic pregnancies occur in the fallopian tubes, most commonly as a result of tubal pathology. Recognized risk factors include a history of pelvic infection, previous surgery, prior ectopic pregnancy, intrauterine device use, and hormonal contraception [3, 4]. Implantation most frequently occurs in the ampulla (70%) and fimbria (11%) [5, 6]. EP is the leading cause of maternal mortality during the first trimester [7] and requires prompt ultrasound diagnosis for timely intervention to prevent severe morbidity [8, 9].

The increasing use of assisted reproductive technologies (ART) has also contributed to a higher incidence of EP [10, 11]. Standard management includes laparoscopic salpingectomy, salpingostomy, or systemic/local methotrexate [12, 13]. In addition, uterine artery embolization (UAE) has gained attention as a minimally invasive method to control hemorrhage, originally established for fibroid treatment [14, 15]. UAE involves the transcatheter injection of embolic agents to occlude uterine arteries, leading to ischemia of the target tissue. For fibroids, this achieves symptom improvement in over 90% of women [15].

While surgical approaches remain first‐line for tubal or ovarian EP, management of cervical and cesarean‐scar pregnancy (CSP) is more complex due to the high risk of life‐threatening hemorrhage. Alternative strategies such as UAE and/or methotrexate may be required [16]. CSP, in particular, is a rare form of EP in which implantation occurs at a uterine incision site with a thin myometrium and marked vascularity, predisposing to rupture and massive bleeding [17]. Moreover, placenta accreta spectrum (PAS) may develop in the context of CSP, further increasing risks and necessitating multidisciplinary care [18].

Evidence suggests UAE can be a safe and effective adjunct or alternative in selected cases. A South Korean series demonstrated high success in women with refractory EP [19], while a U.S. case report confirmed fertility preservation after UAE [20]. A Chinese study showed improved outcomes when UAE was combined with methotrexate [21]. A multicenter study further reported that UAE with absorbable agents, including gelatin sponge (GELFOAM), achieved high technical and clinical success [22]. A recent report from Pakistan described successful fertility preservation after UAE with GELFOAM in broad‐ligament EP [23].

### 1.1 Rationale of the Present Study

Despite these reports, consensus on optimal treatment for cervical and cesarean‐scar EP is lacking. To our knowledge, this is the first Iranian case series to evaluate UAE using GELFOAM exclusively in these rare conditions. Our series contributes by providing one of the largest patient cohorts reported to date and by examining the role of a cost‐effective, absorbable embolic agent within the context of fertility‐preserving management.

## 2 Methods

This descriptive cross‐sectional case series was conducted at the Razi Specialized Vascular Surgery Center, Guilan University of Medical Sciences (Rasht, Iran), between 2021 and 2022. Ethical approval was obtained from the Ethics Committee of Guilan University of Medical Sciences (Approval No: IR.GUMS.REC.1402.140). Written informed consent was obtained from all participants before inclusion.

### 2.1 Participants and Inclusion Criteria

Women presenting with abdominal or pelvic pain, vaginal bleeding, or spotting with positive pregnancy tests were included. Diagnosis of ectopic pregnancy located in the cervix or cesarean‐scar incision was confirmed by ultrasound and serial β‐hCG measurements.

### 2.2 Exclusion Criteria

Women with renal impairment, uncontrolled hypertension, known allergy to contrast agents, or uncontrolled hyperthyroidism were excluded.

### 2.3 Procedural Details

UAE was performed by an interventional radiologist under local anesthesia and sedation. A 5 F catheter was introduced via the femoral artery, and the uterine arteries were selectively catheterized. Absorbable gelatin sponge particles (GELFOAM, Pfizer, USA) cut into 1–2 mm pledgets were injected until near‐stasis of arterial flow was observed. GELFOAM is a temporary embolic material that is resorbed within 4–6 weeks, allowing uterine reperfusion. The average dose was 1–2 vials per patient.

### 2.4 Variables and Data Collection

The following data were extracted from medical records: maternal age, gestational age, baseline and follow‐up β‐hCG levels, size of ectopic mass, obstetric history (gravida, para, previous cesarean or vaginal delivery, miscarriage), and treatment modality.

- Maternal age, gestational age, and β‐hCG levels are summarized in Table 1.
- Obstetric history characteristics are summarized in Table 2.

**Table 1:** Maternal age, gestational age, and β‐hCG levels in patients undergoing UAE with GELFOAM.

Variable	*n*	Min	Max	Median (IQR)	Mean ± SD	Unit
Age	41	25	42	35 (32–38)	35.07 ± 4.40	Years
Gestational age	41	4	10	6 (5–7)	5.97 ± 1.15	Weeks
Primary β‐hCG	39	12	170,773	1000 (1020–38,811)	29,454.87 ± 39,799.28	IU/L
Secondary β‐hCG	37	104	272,000	8377 (3026–34,050)	32,320.22 ± 58,043.21	IU/L
Post‐treatment β‐hCG	9	416	33,211	7010 (2838–25,765)	12,289.44 ± 12,626.05	IU/L

*Note:* Continuous variables are expressed as mean ± SD and median (IQR). Gestational age reported in weeks; β‐hCG in IU/L.

**Table 2:** Obstetric history of patients with cervical and cesarean‐scar ectopic pregnancies.

Variable	0	1	2	3	4	5	6	*n* (%)
Gravida (G)	—	2 (4.9%)	7 (17.1%)	11 (26.8%)	17 (41.5%)	3 (7.3%)	1 (2.4%)	41 (100%)
Para (P)	2 (4.9%)	13 (31.7%)	21 (51.2%)	4 (9.8%)	1 (2.4%)	—	—	41 (100%)
Previous cesarean delivery	18 (43.9%)	13 (31.7%)	8 (19.5%)	2 (4.9%)	—	—	—	41 (100%)
Vaginal delivery	18 (43.9%)	20 (48.8%)	3 (7.3%)	—	—	—	—	41 (100%)
Miscarriage	—	—	—	—	—	—	—	41 (100%)*

*Note:* Gravida = number of pregnancies; para = number of deliveries ≥ 20 weeks; data are presented as frequency (%). Percentages may not sum exactly to 100% due to rounding.

### 2.5 Outcome Definitions

The primary outcome was uterine preservation following UAE. Secondary outcomes included treatment failure and complications (procedure‐specific such as arterial rupture, dissection, pseudoaneurysm, vasospasm; and non‐specific such as pain, infection, or hematoma).

### 2.6 Statistical Analysis

Continuous variables are expressed as mean ± standard deviation (SD) when normally distributed, and as median with interquartile range (IQR) when skewed. Categorical variables are presented as frequencies and percentages. The primary success rate (40/41) is reported with its 95% Wilson confidence interval. Data were analyzed with SPSS version 22 (IBM Corp., Armonk, NY, USA).

## 3 Results

A total of 41 women with ectopic pregnancy located in the cervix or cesarean‐scar incision underwent UAE using absorbable gelatin sponge (GELFOAM).

### 3.1 Baseline Characteristics

The mean age of patients was 35.07 ± 4.40 years (range 25–42). The mean gestational age at diagnosis was 5.97 ± 1.15 weeks (range 4–10). Median baseline β‐hCG was 1000 IU/L (IQR 1020–38,811). Full baseline demographic and laboratory data are summarized in Table 1.

### 3.2 Obstetric History

Most patients were multigravida, with 41.5% having four previous pregnancies. More than half (51.2%) had two prior deliveries, while 43.9% had undergone at least one cesarean section. Detailed obstetric histories are provided in Table 2.

### 3.3 Treatment Outcomes

Successful uterine preservation was achieved in 40 of 41 patients (97.6%). The single treatment failure (2.4%) occurred in a woman with delayed diagnosis; despite UAE, she developed severe postpartum hemorrhage requiring total abdominal hysterectomy.

The 95% Wilson confidence interval for uterine preservation was 87.4%–99.6%.

### 3.4 Complications

No procedure‐specific complications (arterial rupture, dissection, pseudoaneurysm, fistula, vasospasm) were observed. One patient (2.4%) developed a non‐specific postoperative pelvic hematoma, which resolved with conservative management. No infections, thromboembolic events, or major peri‐procedural morbidities were reported.

### 3.5 Treatment Modalities

In addition to UAE, some patients required adjunctive interventions: curettage (58.5%), combined curettage and hysteroscopy (14.6%), wedge resection surgery (17.1%), and wedge resection with laparotomy (4.9%).

## 4 Discussion

The present study demonstrates that UAE with absorbable gelatin sponge (GELFOAM) is an effective and fertility‐preserving treatment for cervical and cesarean‐scar ectopic pregnancies, achieving uterine preservation in 97.6% of cases. These findings are consistent with earlier reports from South Korea, the United States, China, and Pakistan that showed high rates of technical and clinical success using UAE either alone or in combination with methotrexate [19, 21, 23].

Compared to systemic methotrexate, which may require prolonged follow‐up and carries the risk of incomplete resolution, UAE provides immediate control of bleeding and rapid stabilization [12, 13]. Surgical approaches, including wedge resection or hysterectomy, remain necessary in selected cases but are associated with higher morbidity and loss of fertility [16]. In our series, only one patient required hysterectomy due to delayed diagnosis, underscoring the importance of early detection.

[17] Placenta accreta spectrum (PAS) is an important consideration in the context of CSP. PAS is associated with a markedly increased risk of hemorrhage and maternal morbidity, and awareness of this possibility is essential for multidisciplinary planning [17]. The inclusion of PAS in counseling and risk stratification helps guide the choice between conservative and surgical interventions.

The use of absorbable embolic agents such as GELFOAM offers practical advantages: low cost, wide availability, and spontaneous resorption within 4–6 weeks, which allows reperfusion of the uterus and may help preserve fertility [14, 15, 24]. However, superiority over other absorbable materials has not been conclusively established, and thus, UAE should be viewed as part of a broader spectrum of interventional options rather than a universally superior technique [22].

Our complication profile was favorable, with no procedure‐specific adverse events observed. This aligns with prior studies that reported low complication rates, most of which were minor and self‐limiting [19, 24]. One patient developed a postoperative hematoma, which resolved conservatively, indicating that nonspecific complications can occur but are manageable.

The present study adds to the limited literature by providing one of the largest single‐center cohorts treated with UAE for cervical and cesarean‐scar pregnancies. It highlights the feasibility of performing UAE as a first‐line, fertility‐preserving approach in selected patients, especially in settings where surgical risks are high.

### 4.1 Limitations

This study is limited by its single‐center design, modest sample size, and short follow‐up duration. Obstetric outcomes and long‐term fertility after UAE could not be fully assessed. Future multicenter prospective studies with larger sample sizes and longer follow‐up are required to confirm the safety and efficacy of this approach.

## Author Contributions

**Hossein Hemmati:** conceptualization, investigation. **Roya Faraji Darkhaneh:** writing – original draft, software, conceptualization. **Mohaddseh khaligh Dargahi:** writing – review and editing, writing – original draft, data curation. **Mohaya Farzin:** writing – original draft, writing – review and editing, data curation. **Sara Sharifnia:** writing – original draft, data curation, writing – review and editing. **Habib Eslami Kenarsari:** formal analysis, writing – review and editing, writing – original draft. **Zoubin Souri:** supervision, conceptualization, project administration.

## Funding

The authors received no specific funding for this work.

## Ethics Statement

This study was approved by the Ethics Committee of Guilan University of Medical Sciences (Approval no.: IR.GUMS.REC.1402.140). Written informed consent was obtained from all participants prior to inclusion.

## Conflicts of Interest

The authors declare no conflicts of interest.

## Transparency Statement

The lead author Zoubin Souri affirms that this manuscript is an honest, accurate, and transparent account of the study being reported; that no important aspects of the study have been omitted; and that any discrepancies from the study as planned (and, if relevant, registered) have been explained.

## Data Availability

The data supporting the findings of this study are available from the corresponding author upon reasonable request.
